# Antioxidant Activity and Fatty Acid Profile of Sous-Vide Beef Marinated with Kiwiberry Fruit Pulp: Effects of Level Addition and Refrigerated Storage

**DOI:** 10.3390/foods13101446

**Published:** 2024-05-08

**Authors:** Gabriela Haraf, Zuzanna Goluch, Mirosława Teleszko, Piotr Latocha

**Affiliations:** 1 Department of Food Technology and Nutrition, Faculty of Production Engineering, Wroclaw University of Economics and Business, 53-345 Wrocław, Poland; zuzanna.goluch@ue.wroc.pl (Z.G.); miroslawa.teleszko@ue.wroc.pl (M.T.); 2 Department of Environmental Protection and Dendrology, Faculty of Horticulture, Institute of Horticultural Sciences, Warsaw University of Life Sciences, 02-787 Warsaw, Poland; piotr_latocha@sggw.edu.pl

**Keywords:** minikiwi, cooked meat, antioxidant activity, FRAP, ABTS, fatty acid profile, lipid indices, polyphenols, chlorophylls, carotenoids

## Abstract

The purpose of the study was to determine the antioxidant activity (AA) and fatty acid (FA) profile of sous-vide beef previously marinated in brine with a 10, 20 and 30% addition of kiwiberry (*Actinidia arguta* cv. ‘Ananasnaya’) fruit pulp, as well as changes in the parameters studied after 0, 1, 2 and 3 weeks of refrigerated storage in a vacuum package. The FA profile, FRAP (ferric-reducing antioxidant power assay), ABTS (2,2′-azinobis (3-ethylbenzthiazoline-6-acid)), total polyphenols, chlorophylls and carotenoids were also determined in the fruit pulp. Lipid indices for meat were calculated based on the obtained FA profile. The values of FRAP and ABTS of experimental meat products were significantly (*p* ≤ 0.05) higher than those of control samples but decreased with storage time. The proportion of unsaturated FA in the lipids of sous-vide meat was higher in samples with pulp than in control samples and insignificantly decreased with storage time. Meat marinated with kiwiberry pulp was characterized by a significantly (*p* ≤ 0.05) higher proportion of ALA (α-linolenic acid) and LA (linoleic acid), considerably affecting the more favorable value of polyunsaturated FA/saturated FA ratio. A troubling finding was the heightened level of palmitic acid (C16:0) in the lipids of beef subjected to 30% kiwiberry pulp, a factor recognized to play a significant role in the development of various diseases. Beef marinated with 20% kiwiberry pulp addition provides greater nutritional and health benefits than other sample variants because of optimal AA and FA profile changes during refrigerated storage.

## 1. Introduction

Antioxidants are compounds that protect cells and tissues from the adverse effects of oxygen-free radicals, which contribute to the development of diabetes, heart disease and cancer in the human body. The primary sources of antioxidant compounds are vegetables and fruits, including kiwiberry (*Actinidia arguta*), also known as minikiwi, hardy kiwifruit or Bower Actinidia. The polyphenolic compounds it contains limit the formation of oxidative stress in the body. They exhibit a broad spectrum of biological activity, including anti-inflammatory and antimicrobial properties, and have anti-cancer potential [1,2,3,4,5]. A high intake of products that are a source of compounds with antioxidant activity (AA) [6] has been identified as a dietary modifiable factor in reducing the risk of cardiovascular disease. Moreover, a low supply of such products in the diet contributes to increased oxidative stress [5,7].

Antioxidants (endogenous hydrophilic and lipophilic antioxidants) also occur in meat. Among these hydrophilic antioxidant compounds found in meat are enzymes (catalase, glutathione peroxidase), dipeptides (carnosine and anserine), uric acid, amines, selenium and zinc. Lipophilic antioxidants include, among others, α-tocopherol, carotenoids and ubiquinone [8,9]. The AA of meat is much lower than fruits and vegetables, so it is not a good source of antioxidants in the diet. Marinating meat in marinades with a high content of antioxidants could increase its AA. Numerous scientific studies have demonstrated the vital role of marinade ingredients, such as polyphenols, in exerting various beneficial activities in humans, including anti-inflammatory, antibacterial, anti-allergic, antithrombotic, hepatoprotective, antiviral, cardioprotective, anticarcinogenic and vasodilatory effects [10].

Codex Alimentarius Commission authorizes the use of artificial antioxidants in meat processing [11,12]. However, today’s consumers with healthy lifestyles are aware of the possible adverse effects of excessive consumption of artificial compounds. Therefore, they are looking for foods that contain natural antioxidants, such as those derived from fruits [13,14].

At the same time, fat intake and the amount and composition of fats are associated with the risk of developing metabolic diseases [5]. Saturated fatty acids (SFA) are associated with increased serum cholesterol. That is why the emphasis has been placed on reducing the intake of SFAs and increasing the intake of n-3 polyunsaturated fatty acids (PUFA). Indeed, epidemiological and clinical data support the beneficial effect of substituting SFAs with PUFAs [15,16]. In ruminants, microbial breakdown of PUFAs occurs in the rumen, resulting in a smaller amount of PUFAs available for absorption and a larger amount of SFAs and monounsaturated fatty acids (MUFA) in meat [17]. Therefore, efforts are being made to increase the PUFA content of beef [18,19].

The lipids of kiwiberries (located mainly in seeds) contain large amounts of linolenic acid (C18:3n-3), which is valuable from a nutritional point of view [20]. It is the main representative of the n-3 family of fatty acids (FA), known for their health-promoting attributes [21,22,23,24]. These fruits are also characterized by high AA and health properties [25,26]. Although kiwiberry production is still new, its volume constantly grows in many countries, and the raw material is becoming increasingly available [27]. Therefore, in this study, it was hypothesized that marinating developed from brine and kiwiberry fruit could lead to a higher nutritional and antioxidant quality of sous-vide processed beef.

The chosen thermal treatment method (sous vide) involves cooking food products vacuum-sealed in a plastic bag in a water or steam bath at a relatively low temperature (below 100 °C). Chefs at top restaurants began incorporating sous-vide cooking into their culinary techniques during the 1970s. In recent years, particularly in the late 2000s and early 2010s, the use of sous vide has become increasingly common. Sous vide ensures the efficient transfer of heat to food; helps to preserve the food’s shelf-life; and prevents recontamination, off-flavors and flavor loss during the cooking process. Limited access to oxygen and low thermal processing temperature reduces the loss of nutritional values and fat oxidation [28,29]. Some of the research also indicates the beneficial influence of the sous-vide method on the AA of some vegetables compared to traditional cooking [28,30].

The purpose of this study was to determine the AA and FA profile of sous-vide beef previously marinated in brine with 10, 20 and 30% addition of kiwiberry fruit pulp, as well as changes in the parameters studied after 0, 1, 2 and 3 weeks of refrigerated storage compared to control samples marinated in brine only. 

## 2. Materials and Methods

### 2.1. Chemicals

Trolox (6-hydroxy-2,5,7,8-tetramethylchroman-2-carboxylic acid) (Sigma-Aldrich, Steinheim, Germany), ABTS (2,20-azinobis-(3-ethylbenzthiazoline-6-sulfonic acid) (Sigma-Aldrich, Steinheim, Germany), potassium persulfate (Sigma-Aldrich, Steinheim, Germany), acetic acid (Sigma-Aldrich, Steinheim, Germany), TPTZ (2,4,6-tripyridyl-1,3,5-triazine) (Sigma-Aldrich, Steinheim, Germany), ferric chloride (FeCl_3_) (Sigma-Aldrich, Steinheim, Germany), methanol, boron trifluoride (BF_3_) (Sigma-Aldrich, Steinheim, Germany), Supelco 37 FAME Mix C4–C24 Component (Sigma-Aldrich, Steinheim, Germany), Hydrochloric acid (HCl) (Chempur, Piekary Śląskie, Poland), chloroform (Chempur, Piekary Śląskie, Poland), potassium hydroxide (KOH) (Chempur, Piekary Śląskie, Poland), sulphuric acid (H_2_SO_4_) (Chempur, Piekary Śląskie, Poland), Folin–Ciocalteu reagent (Chempur, Piekary Śląskie, Poland), sodium carbonate (Na_2_CO_3_) (Chempur, Piekary Śląskie, Poland), sodium sulfate (Na_2_SO_4_) (Chempur, Piekary Śląskie, Poland), butylated hydroxytoluene (BHT) (AppliChem, Darmstadt, Germany).

### 2.2. Sample Preparing and Sous-Vide Treatment

**Figure 1:**
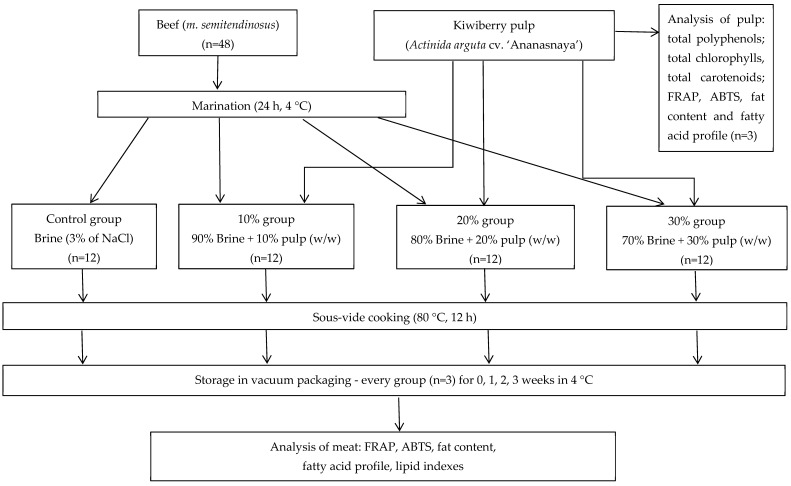
Experiment organization (ABTS—2,2′-azinobis (3-ethylbenzthiazoline-6-acid); FRAP—ferric-reducing antioxidant power assay).

The raw material for the study was beef meat (Eye of Round—false tenderloin) (*m. semitendinosus*) purchased from a local market (Wroclaw, Poland). The culinary elements purchased were vacuum-packed and came from the same manufacturer and production batch. Forty-eight portions (slices) of beef (approximately 100–120 g each) were assigned to 4 groups (12 samples in each group) (Figure 1). Kiwiberry fruits (*A. arguta* cv. ‘Ananasnaya’) came from the experimental field of the Department of Environmental Protection, Warsaw University of Life Sciences. The pulp was obtained by homogenizing the kiwiberry fruits with the peel (IKA T25 Ultra-Turrax, IKA-Werke GmbH & CoKG, Staufen im Breisgau, Germany). The beef slices were dipped in the marinades in a glass dish covered with cling film and stored in the refrigerator at 4 °C for 24 h. The scheme of the experiment and the composition of the marinades are shown in Figure 1. After marinating, the meat pieces were taken out of the marinade, then gently cleaned of the excess marinade with a knife, and briefly dried with a paper towel. Next, meat samples were vacuum packed using a vacuum chamber packaging machine (Profi Line 410, Hendi BV, De Klomp, The Netherlands) in bags for sous-vide cooking (Hendi BV, 75 μm thickness). Packaged meat samples were subjected to thermal treatment in a water bath with automatically controlled water temperature (Aquarius L100/150/200, Warsaw, Poland) for 12 h at 80 °C. The sensory properties of the muscle determined thermal treatment parameters. Eye of Round (*m. semitendinosus*) is a meat with low tenderness, requiring a higher temperature and longer processing time [28,29]. The time and temperature of the heat treatment in the experiment were determined experimentally to obtain a ready-to-eat product with appropriate organoleptic parameters. After heat treatment, meat from the control and experimental groups was divided into 4 groups and stored under refrigeration for different lengths of time: 24 h (storage 0, n = 3), 1 week (n = 3), 2 weeks (n = 3) and 3 weeks (n = 3). The samples were stored in the same package in which they were heat-treated, and the applied maximum storage time resulted from previously conducted (unpublished) microbiological tests. Total microbial counts began to increase in the control sample after 6 weeks of chilled storage (4 °C). However, the average temperature in home refrigerators is about 7 °C [31], and in such a situation, the storage time, for safety reasons, should be reduced by 50% [32]. Because the research was intended to mimic consumer behavior, the 3-week storage period was established. After each storage period, the meat was removed from the vacuum packs and, after gently drying the surface with a paper towel, ground in a meat grinder and freeze-dried (Alpha 1–4 LSCplus, Martin Christ, Osterode am Harz, Germany). The lyophilization conditions are as follows: product input temperature: −75 °C; shelf temperature: 25 °C; vacuum: 0.2 mbar; time: 21 h. After freeze-drying, samples were stored at −80 °C until further analysis.

### 2.3. Preparation of Fruit Pulp Samples for AA Determination

The kiwiberry pulp was lyophilized (see Section 2.2) and used to prepare extracts, in which the content of polyphenolic compounds and antioxidant activity were determined. The extracts were prepared from about 0.5000 g of lyophilisate and poured into 10 cm^3^ of 80% methanol acidified with 1% addition of concentrated HCl. They were then sonificated for 15 min (Sonic 6D, Polsonic, Warsaw, Poland), after which they were left for 24 h at 4 °C (refrigerator). After this time, the extracts were again subjected to ultrasound for 15 min (40 kHz, ±20 °C). After sonification, the samples were centrifuged in a refrigerated centrifuge (MPW-380R; MPW Med. Instruments, Warsaw, Poland) for 10 min at 4 °C at 10,000 rpm. The resulting supernatant was poured into 10 cm^3^ PET tubes. The resulting extracts were stored at −20 °C in a freezer until FRAP and ABTS analyses, the course of which is described in Section 2.6 and Section 2.7.

### 2.4. Determination of Total Polyphenols (TP) in Kiwiberry Pulp by Folin–Ciocalteu Method

The TP in pulp extract was determined using the method described in [33] with minor modifications. Fruit pulp extracts (0.1 cm^3^) were taken into spectrophotometric cuvettes, and then 0.2 cm^3^ of Folin–Ciocalteu reagent, 2 cm^3^ of distilled water and 1 cm^3^ of 15% Na_2_CO_3_ solution were added. The cuvettes with the obtained reaction mixture were placed in a laboratory cabinet for 60 min (temperature ± 20 °C, no light access). Next, the absorbance of the tested samples was read in a spectrophotometer (UV 1900i, Shimadzu, Tokyo, Japan) at a wavelength of 765 nm. Absorbance results were converted to mg GA (gallic acid)/100 g FM of the sample.

### 2.5. Total Chlorophylls (TChl) and Carotenoids (TC) Determination in Kiwiberry Pulp

To determine the TChl (as the sum of chlorophyll a and b content) and TC [34], ±1.0000 g of lyophilized pulp were weighed into 15 cm^3^ centrifuge tubes. Then, 10 cm^3^ of methanol (HPLC grade) was added. The samples were shaken manually for 5 min, left for extraction for 2 h in the dark and then centrifuged for 10 min at 10,000 rpm in a refrigerated centrifuge (MPW 350R, MPW Instruments, Warsaw, Poland). The absorbance of the methanol extracts prepared in this way was measured at three wavelengths: 652.4 nm, 665.2 nm and 470 nm in a UV 1900i Shimadzu spectrophotometer (Tokyo, Japan). The results are given in mg of phytochemical/100 g of the pulp’s FM.

### 2.6. Preparation of Meat Samples for AA Analysis

Meat extracts used to determine antioxidant activity (FRAP, ABTS) were prepared by mixing lyophilized samples (approx. 0.20000 g) with 5 cm^3^ of phosphate buffer (pH = 6.8). The mixture was shaken manually for 5 min and then left for 24 h at 4 °C. Then, the samples were centrifuged in a refrigerated centrifuge (MPW-380R; MPW Med. Instruments, Warsaw, Poland) for 10 min at 4 °C at a speed of 10,000 rpm. The obtained supernatant was filtered using 0.45 μm nylon syringe filters into 15 cm^3^ PET tubes.

### 2.7. The AA Analysis of Kiwiberry Pulp and Meat Using the FRAP Method

The analysis was performed according to [35]. The FRAP reagent was prepared by mixing acetate buffer pH 3.6 (300 μmol), 10 μmol TPTZ in 40 μmol HCl, and 20 μmol FeCl_3_ in a ratio of 10:1:1 (*v*/*v*/*v*). Tested extracts (0.5 cm^3^) were placed in spectrophotometric cuvettes, and 0.5 cm^3^ of redistilled water and 3 cm^3^ of FRAP solution were added and mixed thoroughly. After 10 min, the absorbance of the solution was measured at a wavelength of 593 nm against redistilled water (Shimadzu, UV-1900i, Kyoto, Japan). The analysis results were expressed in μmol of Trolox (TE)/100 g of sample.

### 2.8. The AA Analysis of Kiwiberry Pulp and Meat Using the ABTS Method

The determination was performed using the method described in [36]. The starting solution of ABTS^•+^ cation radical, containing 7 mmol ABTS^•+^ and 2.45 mmol potassium persulfate, was diluted with redistilled water before analysis so that at λ = 734 nm, its absorbance was ~0.7. 0.03 cm^3^ of the test extracts (or redistilled water in the case of a blank) were taken into spectrophotometer cuvettes, and 3 cm^3^ of ABTS^•+^ solution was added. After 6 min, the absorbance of the samples (λ = 734 nm) was measured against the blank (Shimadzu, UV-1900i, Kyoto, Japan). The analysis results were expressed in mmol Trolox (TE)/100 g of fruit pulp or meat.

### 2.9. Crude Fat Analysis

The kiwiberry pulp and meat were tested for total fat (Soxhlet procedure; Automatic Soxhlet Extractor SOX606; Hannon Instruments, Jinan, China) with the AOAC [37] method (960.39). 

### 2.10. FA Profile Analysis of Kiwiberry Pulp and Meat (GC-FID Method)

Lipids of kiwiberry pulp were extracted by homogenization of ±5.0 g of lyophilized pulp with the mixture of chloroform and methanol (2:1; *v*/*v*) containing 0.001% of BHT as an antioxidant. The solvent was then evaporated in a nitrogen stream. Next, the crude lipid extract was mixed with 0.5 M methanolic solution of KOH. According to the Ce 2.66 official method [38], the BF_3_ (boron trifluoride) solution in methanol was used for FA transesterification.

Fatty acid profile determination of meat was performed by a one-step extraction–transesterification method [39,40]. A lyophilized meat sample (0.1 g) was added to 2 cm^3^ of a mixture of methanol and sulfuric acid (85:15, *v*/*v*) and 2 cm^3^ of chloroform. The samples were heated for 30 min at 100 °C. In the next step, after the samples cooled to room temperature, 1 cm^3^ of distilled water was added and stirred for about 1 min. Then, after the phases separated, the lower phase was taken into test tubes with Na_2_SO_4_ to dry. 

Gas chromatography analysis was performed just after sample preparation. The chromatographic separation conditions followed the procedure of [41]. The FA in the form of methyl esters (FAME) were quantified by GC (Agilent 7890 A series, Agilent Tech. Inc., St. Clara, CA, USA), coupled with FID (flame-ionization detector), and fused silica capillary column J&W Scientific HP-88 series (length: 100 m; diameter: 0.25 mm; film: 0.20 µm; Agilent Tech. Inc., St. Clara, CA, USA). FA peaks were identified by comparing retention times to a mixture of external standard methyl esters (Supelco 37 FAME Mix C4–C24 Component, Sigma-Aldrich, St. Louis, MI, USA). The results are presented as the percentage of individual FA in the sum of all FA. 

### 2.11. Health Lipid Indices Calculation

Based on the shares of particular FA in lipids of the sous-vide beef (regardless of storage), the following health lipid indices were calculated (full names of FA are provided in Tables 6, 7 and 8):

1. PUFA/SFA = sum of the PUFA determined/sum of the SFA determined;
2. P/S = (C18:2n-6 + C18:3n-3)/(C12:0 + C14:0 + C16:0) [42];
3. LA/ALA = C18:2n-6/C18:3n-3;
4. n-6/n-3 = sum of the PUFA n-6 determined/sum of the PUFA n-3 determined
5. Atherogenicity index AI = [C12:0 + (4 × C14:0) + C16:0]/[ΣMUFA + ΣPUFA n-6 + ΣPUFA n-3] [43];
6. Thrombogenicity index IT = (C14:0 + C16:0 + C18:0)/(0.5 × MUFA) + (0.5 × PUFA n-6) + (3 × PUFA n-3) + (PUFA n-3/PUFA n-6) [43];
7. Hypocholesterolemic FA/Hypercholesterolemic FA ratio h/H = [(C18:1 n-9 + C18:2n-6 + C18:3n-3 + C20:3n-6 + C20:4n-6 + C20:5n-3 + C22:5 n-3, C22:6 n-3)/(C14:0 + C16:0)] [44].

The atherogenicity index (AI) indicates the relationship between the sum of the main proatherogenic SFAs and that of the main classes of antiatherogenic UFA. The FAs, favoring the adhesion of lipids to cells of the immunological and circulatory systems, are considered proatherogenic. The antiatherogenic FAs inhibit plaque aggregation and diminish the esterified FA levels, phospholipids and cholesterol, thereby preventing the appearance of microcoronary and macrocoronary diseases. The thrombogenicity ratio (TI) is defined as the relationship between the prothrombogenic (SFAs) and antithrombogenic FAs (MUFAs, n-3 and n-6 PUFAs); it shows a tendency to form clots in the blood vessels [45]. The P/S is the ratio of the sum of LA (C18:2n-6) and ALA (C18:3n-3) (precursors from which the remaining n-3 and n-6 PUFA are produced) to the shares of acids recognized as the most harmful to health: C12:0, C14:0 and C16:0.

### 2.12. Statistical Analysis

The data were analyzed using Statistica 13.1 software (StatSoft Inc., Tulsa, OK, USA) as a completely randomized design using the following:

1. Two-way ANOVA concerning the kind of marinade (control, 10%, 20% and 30% fruit addition) and time of storage (0, 1, 2 and 3 weeks) as a factorial design (4 × 4), according to the following linear model: Y_ij_ = μ + A_i_ + B_j_ + (AB)_ij_ + e_ij_, where Y_ij_ = value of trait (the dependent variable); μ = overall mean; A_j_ = effect of marinade kind; B_j_ = effect of time of storage; (AB) = interaction; e_ij_ = random observation error;
2. One-way ANOVA according to the following linear model Y_ij_ = μ + A_j_ + e_ij_, where Y_ij_—value of trait; μ—overall mean; A_j_—effect of marinade; e_ij_—random observation error.

The statistical significance of differences between the averages of the groups was calculated using Tukey’s multiple comparisons test, on the level of significance *p* ≤ 0.05, using Statistica 13.1 software. The tables show arithmetic means and standard errors of the means (SEM). The results are the average of determinations performed on three samples (n = 3).

## 3. Results and Discussion

### 3.1. Antioxidant Properties of Kiwiberry Fruit Pulp

In the current study, we evaluated the content of selected phytochemicals and the AA of pulp from kiwiberry fruit (cv ‘Ananasnaya’). Table 1 shows the characteristics of the pulp concerning the TP, TChl and TC, and AA, as estimated by FRAP and ABTS methods. As mentioned in the Materials and Methods subsection, the marinades were a mixture of water, salt and homogenized kiwiberries. Hence, the fruit was the only source of substances with antioxidant potential. As the fruit content of the marinade increases, the content of phytochemicals and the value of AA also increases. On a dry mass basis, the TP in fruit pulp was 951.63 mg GA/100 g DM, and the AA was 10.00–15.01 mmol TE/100 g DM. According to other authors [46], the TP of kiwiberry fruit can be as high as 6679.18 mg/100 g DM, and its AA measured by the ABTS method is in the range of 9.40–21.36 mmol TE/100 g DM. Another experiment [47] revealed a lower TP content of 101.9–106.1 mg of GA/100 g FM determined in *A. arguta* cultivar ‘Ananasnaya’ than in the present study.

Polyphenols are a vital group of antioxidants of plant origin. The AA of polyphenols is due to the presence of hydroxyl groups in the structure of these compounds. It has been shown to depend on the number of these groups and their position in the molecule. In the case of flavonoids, the mechanism involves the uptake of oxygen free radicals and their reactive forms (RFTs) and the inhibition of the activity of enzymes involved in their formation. Indirectly, flavonoids can chelate transition metal ions and protect low-molecular-weight antioxidants. Such properties of flavonoids make these compounds important in combating and preventing various conditions accompanied by oxidative stress, including inflammatory processes, type 2 diabetes, atherosclerosis, neurodegenerative diseases or cancer [5]. In the case of phenolic acids, the antioxidant potential is a result of the reducing nature of the molecule; the ability to bind free radicals, chelate metal ions of enzymes catalyzing oxidation reactions and interrupt radical chain reactions; and the ability to inhibit oxidases [48,49,50,51].

The TChl could also partially affect the AA of kiwiberry pulp (Table 1). According to the study [52], the green pigment concentration in the fruit of *A. arguta* ranges from 2.6 to 4.2 mg/100 g FM. Other authors [53,54] also determined the TChl of several kiwiberry cultivars, reporting a range of 2.28 to 8.02 mg/100 g FM [53,54]. The structure and configuration of chlorophylls affect their AA. However, an analysis of the literature shows no clear opinion on the differences in AA between chlorophylls a and b. For example, some of the authors [55,56], showed that chlorophyll b compounds have higher AA than chlorophyll a, suggesting an unknown role for the aldehyde group at the C7 position in shaping the antioxidant capacity of these compounds. Others [57] came to a different conclusion, proving that chlorophyll a is three times more effective as a radical quencher than chlorophyll b [57].

### 3.2. The AA of Sous-Vide Beef Marinated with Kiwiberry Fruit Addition

Table 2 shows the results of the analysis of the AA of beef measured by the FRAP method, taking into account the type of marinade and the storage time. Samples stored in different marinades differed in AA; however, the differences were not statistically significant in all cases. Samples marinated with 30% added kiwiberry showed greater AA than those immersed in brine only and brine with 10% added fruit. The storage time of the meat also significantly (*p* ≤ 0.05) affected its AA. The AA increased in the first week of storage, with a significant decrease in the second and third weeks. Samples marinated with 30% fruit had higher AA than control samples in the 1st, 2nd and 3rd weeks of storage.

The results of the analysis of the AA of sous-vide beef marinated with different percentages of kiwiberry pulp, as measured by the ABTS method, are shown in Table 3. Based on the data obtained, it can be concluded that with a higher percentage of fruit pulp in the marinade, the AA measured by the ABTS method also increased significantly (*p* ≤ 0.05). The FRAP value was also influenced by the time of storage (Table 2). Depending on the sample, the AA reached a maximum in the first or second week of storage, after which it decreased to a value lower than the initial one on the first day after heat treatment.

In conclusion, it was shown that the highest AA measured by FRAP and ABTS methods was in meat marinated with 30% added kiwiberry and stored for one week. In both methods, in general, samples marinated with added fruits showed higher AA, but these values decreased with storage time. The decrease in AA values during storage is a phenomenon often described both in the context of foods of plant origin, e.g., fruit puree [58,59,60] or juices [61,62,63], but also meat, fish and their products [64,65,66]. As mentioned earlier, secondary plant metabolites from the polyphenol group present in the kiwi berry marinade were primarily responsible for the AA of beef. The progressive degradation of these compounds is generally responsible for the decreasing antioxidant potential of the products in which they are present. It is the result of, among other things, processes of oxidation of polyphenols, the formation of their complexes with other food components, enzymatic transformations, an increase in the oxidative potential of the environment, and the transition of the active form to the pro-oxidant form, or changes in pH [67]. Partial oxidation of polyphenols, however, may result in their increased ability to bind free radicals compared to non-oxidized forms, which was the likely cause of the increase in AA during the first week of storage of marinated beef. Such a phenomenon was observed, for example, in the case of enzymatically oxidized catechin, with a higher degree of oxidation already resulting in a loss of AA. The improved free radical binding capacity of partially oxidized polyphenols can be explained by their more remarkable ability to release the hydrogen atom of the hydroxyl group at the aromatic ring and by the increased ability of the aromatic ring to hold unpaired electrons [68].

A decrease in AA measured by ABTS and DPPH with storage time was also observed by other authors [69] in baked pork meat products fortified with lyophilized dragon fruit pulp. In the above-cited study, sample variants with the highest addition of lyophilized fruit also showed the highest AA. Similar results were also obtained [70] in the studies on AA measured by the DPPH value of raw chicken breast marinated with microencapsulation of turmeric extract stored at refrigeration (4 ± 1 °C) for 0, 3, 6, 9 and 12 days decreased with time [70]. A higher addition of turmeric extract resulted in a greater AA value.

However, adding antioxidants to the marinade does not always produce better antioxidant status. The study [71,72] investigated the antioxidant capacity changes in different marinated oven-grilled chicken breast and pork neck meat. The different marination variants involved cranberry and grape pomace and Baikal skullcap, which subsequently incorporated either African spice or industrial marinade/pickle. The ABTS values they obtained ranged from 1.82 to 2.52 mMol Trolox in chicken breast and from 1.80 to 2.29 mMol Trolox in pork neck meat. The pork ABTS, DPPH and FRAP values seemingly decreased with increasing antioxidant concentrations in marinades. The authors observed the absence of a synergistic effect of marinating meat in marinades containing several different substances with high antioxidant potential on the AA of the resulting meat products. The samples were not stored, so the authors stated that understanding this issue appears challenging. In our study, non-stored control samples (0 weeks) for both ABTS and FRAP analysis had higher AA than 10% samples (Table 2 and Table 3). This situation changes after one week of storage, particularly for ABTS values. As mentioned earlier, it is possible that for the full antioxidant potential to be revealed, time is needed for the polyphenols present in the material to undergo partial oxidation, increasing their ability to bind free radicals.

### 3.3. Fat Content and FA Profile of Kiwiberry Fruit Pulp Used in Marinades

The fat content of kiwiberry (*A. arguta* cv. ‘Ananasnaya’) fruit pulp was 0.54 g/100 g FM (Table 4). The study [20] determined a similar fat content in *Actinidia deliciosa* cv. ‘Hayward’ and *Actinidia chinensis* cv. ‘Hort 16A’ (0.52 and 0.56 g/100 g FM, respectively). The lipids of the ‘Ananasnaya’ cultivar were dominated by PUFA (74.87%), the main representative of which was ALA—65.55%) (Table 4). The only representative of MUFA was C18:1n-9cis (oleic acid—15.66%). The proportion of SFA was 9.48%, including 7.3% of C16:0 (palmitic acid) and 2.18% of C18:0 (stearic acid). Comparable results regarding PUFAs were obtained by Jin et al. [73]. In their study, the lipids of three traditional Korean *Actinidia arguta* fruit varieties (Otumsense, Chiak and Skinny Green) were characterized by a PUFA proportion of 67.67–83.9%, with ALA (47.95–69.55%) being the main representative. However, in the study mentioned above, more SFAs were determined (13.91–30.54%), including C22:0 and C23:0 acids, and less C18:1n-9cis acid (1.02–1.56%). In a study by Liang et al. [74], the lipids of wild Chinese *A. arguta* were characterized by a slightly higher proportion of ALA (77.78%) and comparable proportions of LA (10.57%), C16:0 (6.66%) and C18:0 (3.09%). In addition, the studies of these authors also identified the presence of C20:0 and C20:1 acids.

### 3.4. Fat Content, Lipid FA Profile and Lipid Indices of Marinated Sous-Vide Beef

Our study showed that adding fruit to the marinade did not significantly affect the fat content of the meat samples, ranging from 3.49 to 3.65 g/100 g FM (Table 5). Samples marinated with 30% added kiwiberry pulp, compared to control samples, were characterized by a higher proportion of SFAs (including C12:0, C13:0 and C16:0) and lower MUFAs, including C18:1cis (Table 6 and Table 7). All samples marinated with fruit pulp had more PUFA than meat marinated only in brine (Table 8). The higher proportion of PUFA could probably have resulted from the higher content of LA (C18:2n-6) and ALA (C18:3n-3), which are components of lipids located mainly in the kiwiberry seeds (9.32 and 65.55%, respectively) (Table 4). Similarly, in the case of saturated FAs, the lipids of samples with 30% added fruit contained more C16:0 acid, which may be a consequence of the presence of this acid in fruit lipids (7.30%). It is not beneficial to consumer health, as studies have found palmitic acid to be a risk factor for metabolic syndrome, cardiovascular disease, cancer, neurodegenerative diseases and inflammation [75,76]. For some unknown reason, despite C18:1n-9 acid in the kiwiberry pulp, a higher proportion of this acid was not observed in samples marinated with fruit compared to the control group.

The FA profile of the beef studied was dominated among SFAs by C16:0 and C18:0 acids, among MUFAs by C18:1c-9, and PUFAs by C18:2n-6. It is consistent with previous results from other authors [17,77,78,79]. In the study [79], cooked beef had similar PUFA content, including a higher proportion of LA (8.32%) and a lower proportion of ALA (0.14%). In addition, the studies cited above had a higher proportion of SFA (50.7%), lower MUFA (39.0%) and a higher n-6/n-3 value (8.35) than in our experiment. On the other hand, in other studies [78], a similar proportion of SFA (42.76%), lower MUFA (31.57%), but higher PUFA (18.77%), including a higher proportion of LA and ALA (10.36 and 2.34%, respectively), was determined in cooked beef [78]. In a study [69], significant differences were noted in the levels of SFA, MUFA and PUFA, including PUFA n-3, in pork products that had various amounts of lyophilized dragon fruit pulp added. Products containing this addition showed higher levels of all these fatty acids, leading to an improved n-6/n-3 ratio [69]. In contrast, in the other study [80], there was no targeted trend of changes in the FA profile of raw mutton marinated in fermented (with lactic fermentation bacteria) sour whey with the addition of lyophilized apple and blackcurrant pomace consisting of peel, pulp and seeds. In this case, the authors conclude that in addition to the marinade ingredients, the FA profiles may have been influenced by lactic fermentation bacteria, which have different lipolytic activity and the capacity to preclude the oxidation of unsaturated free FAs. 

The proportion of PUFA decreased with increasing storage time, but these changes were insignificant within individual marinades (Table 8). The proportion of LA (C18:2n-6) and C22:6n-3 significantly reduced with time. The proportion of C20:4n-6 acid also decreased but not significantly. The relatively most diminutive losses of PUFA were observed in samples with 10 and 20% addition of kiwiberry fruit pulp. Changes in the other acid groups were subtle. The proportion of SFA generally increased, but these changes were not statistically significant (Table 6). Among SFA, the proportion of C10:00 (for 20% and 30% samples) and C12:0 (for samples with 20% pulp addition) increased significantly with time. The proportion of MUFA did not show a uniform change trend with time (Table 7). 

A study on vacuum-packed raw ostrich meat observed a significant decrease in total PUFAs during 16-day storage [81]. In contrast, for vacuum-packed traditional Kazakh dry-cured beef, storage time (30 days) did not significantly affect the proportion of SFA, MUFA and PUFA [82]. Similarly, [83] no significant differences in the FA profile of breast chicken meat stored for nine days in vacuum packaging under refrigerated conditions were found [83]. In the studies above, the sum of PUFAs decreased with storage time, but these differences were not statistically significant. 

Marinating with adding kiwiberry pulp affected PUFA/SFA and P/S positively and favourably but not significantly LA/ALA (Table 5). This is because of the higher content of total PUFAs, LA (C18:2n-6) and especially ALA (C18:3n-3). However, despite the higher content of this n-3 acid in the samples marinated with fruits, there were no significant differences in n-6/n-3. In the case of 30% samples, n-6/n-3 was slightly higher and, therefore, less favorable. Perhaps this is related to the content of C18:2n-6 in kiwiberry seeds, which increased the sum of PUFA n-6 and thus affected the value of this index [45].

The AI and TI indicate potential for stimulating platelet aggregation [84]. Thus, the smaller the AI and TI values, the greater the protective potential for coronary artery disease. Marinating with the addition of fruit pulp did not improve the values of lipid health quality indexes such as AI, TI and h/H compared to control samples. The AI and TI indexes were significantly more favorable for samples with 20% kiwiberry pulp added to the marinade than those with 30% addition. The 30% samples had the highest but the least favorable average h/H ratio. The control samples had significantly more favorable AI and h/H ratios than the 30% samples. The reason was the higher content of C12:0 and C16:0 acids in the marinated samples with fruit pulp.

To prevent ischemic heart disease and cancer, the PUFA/SFA ratio in the diet should be higher than 0.45 [85,86]. In the current study, the value of this ratio is lower than recommended. The P/S index value for the analyzed beef ranged from 0.13 to 0.19 and was more favorable for beef marinated with kiwiberry fruits. 

The diet’s balanced n-3 and n-6 PUFA content reduces the risk of inflammatory diseases, such as cardiovascular disease, diabetes, rheumatoid arthritis, asthma, cancer, etc. [16,87,88]. According to the latest academic updates in the literature, the lower the n-6/n-3 ratio, the better. The optimal ratio may vary with the disease under consideration, but the closer the ratio is to 1:1, the better [87]. The n-6/n-3 ratio below 4 to 5, as in current studies, is close to the values recommended in older publications (below 4.0) [86]. The n-6/n-3 ratios in analyzed beef were higher than recommended, but at the same time, they were lower than in the so-called contemporary Western diet, which is even 16.7:1 [21,22]. Recommended values for AI and TI are less than 1.0 and 0.5, respectively [86]. Therefore, the AI value for the tested beef is within the recommended range but not TI.

## 4. Conclusions

Based on the study, it can be concluded that adding kiwiberry pulp to the marinade significantly increased the AA of sous-vide beef compared to control samples marinated only in 3% brine. However, this activity decreased with increasing storage period. Samples marinated with 20 and 30% fruit pulp addition after one week of storage had the highest FRAP and ABTS values. Sous-vide beef marinated in kiwiberry pulp was characterized by more PUFA in lipids, including LA and ALA. As a result, the meat had significantly more favorable PUFA/SFA and P/S ratios compared to control samples. However, this did not translate into more favorable values for the other calculated lipid indices, i.e., n-6/n-3, AI, TI and h/H. A concerning observation was the increased concentration of palmitic acid (C16:0) in the lipids of beef treated with 30% kiwiberry pulp, a factor known to contribute significantly to the pathogenesis of various diseases. The changes observed during storage in ΣSFA, ΣMUFA and ΣPUFA were not statistically significant, but relatively most considerable PUFA losses were observed in samples of 30%. Because of its better AA and FA profile, consumers can gain the most nutritional and health advantages when choosing sous-vide beef marinated with 20% kiwiberry. Considering the alterations in AA and FA profiles, the optimal storage duration for this beef was one week.

## Figures and Tables

**Table 1:** Content of selected phytochemicals and AA of kiwiberry fruit pulp (n = 3).

Parameter		SEM
TP [mg of GA/100 g FM]	656.62	15.43
TChl [mg/100 g FM]	5.37	0.96
TC [mg/100 g FM]	1.10	0.39
ABTS [mmol TE/100 g FM]	6.97	0.51
FRAP [mmol TE/100 g FM]	10.42	0.75

TP—total polyphenols; TChl—total chlorophylls (sum of chlorophyll a + b); TC—total carotenoids; FM—fresh mass; GA—gallic acid; ABTS—2,2′-azinobis (3-ethylbenzthiazoline-6-acid); FRAP—ferric-reducing antioxidant power assay; TE—Trolox Equivalent; SEM—standard error of the means.

**Table 2:** AA of marinated sous-vide beef measured by the FRAP method.

Storage Time	Control *	Beef Marinated with the Kiwiberry Addition **			SEM
		10%	20%	30%	
0	^z^ 0.15 ^a^	^z^ 0.11 ^b^	^z^ 0.13 ^a^	^z^ 0.14 ^a^	0.003
1 week	^w^ 0.21 ^b^	^w^ 0.22 ^b^	^w^ 0.25 ^a^	^w^ 0.25 ^a^	0.004
2 weeks	^x^ 0.17 ^b^	^y^ 0.19 ^a^	^y^ 0.20 ^a^	^y^ 0.20 ^a^	0.003
3 weeks	^y^ 0.18 ^c^	^x^ 0.20 ^b^	^x^ 0.22 ^a^	^z^ 0.23 ^a^	0.004
Total	0.18 ^b^	0.18 ^b^	0.19 ^ab^	0.20 ^a^	0.004
SEM	0.004	0.007	0.009	0.007	

n = 3 and, for total value, n = 12; *—beef marinated in 3% brine; **—[μmol Trolox (TE)/100 g DM] ^a,b,c^—means with different letters in the same row differ at *p* ≤ 0.05; ^w,x,y,z^—means with different letters in the same column differ at *p* ≤ 0.05; SEM—standard error of the mean.

**Table 3:** AA of marinated sous-vide beef measured by the ABTS method.

Storage Time	Control *	Beef Marinated with the Kiwiberry Addition **			SEM
		10%	20%	30%	
0	^w^ 0.99 ^c^	^x^ 0.83 ^d^	^x^ 1.28 ^b^	^y^ 1.38 ^a^	0.04
1 week	^x^ 0.87 ^d^	^w^ 1.21 ^c^	^w^ 1.44 ^b^	^w^ 1.60 ^a^	0.05
2 weeks	^w^ 0.95 ^c^	^y^ 1.14 ^b^	^w^ 1.48 ^a^	^x^ 1.53 ^a^	0.05
3 weeks	^y^ 0.74 ^b^	^z^ 0.81 ^b^	^y^ 1.00 ^a^	^z^ 1.03 ^a^	0.03
Total	0.89 ^d^	0.99 ^c^	1.30 ^b^	1.39 ^a^	0.02
SEM	0.02	0.03	0.03	0.05	

n = 3 and, for total value, n = 12; *—beef marinated in 3% brine; **—[μmol Trolox (TE)/100 g DM]; ^a,b,c,d^—means with different letters in the same row differ at *p* ≤ 0,05; ^w,x,y,z^—means with different letters in the same column differ at *p* ≤ 0.05; SEM—standard error of the mean.

**Table 4:** Fat content and FA profile of *A. arguta* pulp (with seeds) used in marinades.

Parameter		SEM
Crude fat [g/100 g FM]	0.54	0.09
Percentage of all FA:		
C16:0 (Palmitic)	7.30	0.06
C18:0 (Stearic)	2.18	0.08
C18:1n-9cis (Oleic)	15.66	0.20
C18:2n-6 (LA—linoleic)	9.32	0.16
C18:3n-3 (ALA—α-linolenic)	65.55	0.15
SFA	9.48	0.14
MUFA	15.66	0.20
PUFA	74.87	0.16

n = 3; SFA—saturated fatty acids, MUFA—monounsaturated fatty acid; PUFA—polyunsaturated fatty acids; FM—fresh mass; SEM—standard error of the mean.

**Table 5:** The fat content and lipids nutritional quality indexes of marinated sous-vide beef.

Parameter	Control *	Beef Marinated with the Kiwiberry Addition			SEM
		10%	20%	30%	
Crude fat [g/100 g FM]	3.49	3.55	3.57	3.65	0.04
PUFA/SFA	0.18 ^b^	0.20 ^ab^	0.23 ^a^	0.22 ^a^	0.04
P/S	0.13 ^b^	0.17 ^a^	0.17 ^a^	0.19 ^a^	0.01
LA/ALA	8.69	7.30	7.34	6.74	0.21
n-6/n-3	4.68	3.83	3.72	4.85	0.17
AI	0.87 ^b^	0.88 ^ab^	0.87 ^b^	0.96 ^a^	0.01
TI	1.33 ^ab^	1.32 ^ab^	1.26 ^b^	1.41 ^a^	0.02
h/H	1.70 ^a^	1.69 ^a^	1.76 ^a^	1.54 ^b^	0.03

n = 3; *—beef marinated in 3% brine; ^a,b^—means with different letters in the same row differ at *p* ≤ 0.05; SEM—standard error of the mean. Full names of indexes—see Section 2.11.

**Table 6:** The share of SFA in the lipids of marinated sous-vide beef.

Fatty Acids (% of the Sum of All FA)	Storage Time	Control *	Beef Marinated with the Kiwiberry Addition			SEM
			10%	20%	30%	
C10:0 (Capric)	0	^x^ 0.22	0.20	0.23	0.20	0.01
	1 week	^y^ 0.11 ^b^	0.20	0.22 ^a^	0.16	0.01
	2 weeks	^y^ 0.11	0.16	^y^ 0.17	^y^ 0.13	0.01
	3 weeks	0.12 ^b^	0.24 ^a^	^x^ 0.33 ^a^	^x^ 0.25 ^a^	0.03
	Total	0.14 ^c^	0.20 ^ab^	0.24 ^a^	0.18 ^b^	0.01
	SEM	0.01	0.01	0.02	0.02	
C12:0 (Lauric)	0	0.06 ^b^	0.12 ^b^	^y^ 0.21	0.80 ^a^	0.09
	1 week	0.04	0.27	^y^ 0.35	0.43	0.05
	2 weeks	0.06	0.25	^y^ 0.30	0.60	0.07
	3 weeks	0.31 ^b^	0.38 ^b^	^x^ 1.08 ^a^	0.84	0.13
	Total	0.12 ^b^	0.26 ^b^	0.49 ^a^	0.66 ^a^	0.05
	SEM	0.05	0.04	0.11	0.10	
C13:0 (Tridecanoic)	0	0.05 ^b^	0.01 ^b^	0.04 ^b^	^x^ 0.13 ^a^	0.02
	1 week	0.03	0.06	0.09	^y^ 0.04	0.01
	2 weeks	0.03	0.06	0.07	^y^ 0.05	0.01
	3 weeks	0.00 ^b^	0.04	0.08 ^a^	0.05	0.01
	Total	0.03 ^c^	0.04 ^bc^	0.07 ^a^	0.06 ^b^	0.01
	SEM	0.01	0.01	0.01	0.01	
C 14:0 (Myristic)	0	1.39	1.70	2.84	1.52	0.36
	1 week	1.73	1.56	2.24	2.64	0.22
	2 weeks	2.34	1.82	1.61	2.81	0.20
	3 weeks	2.45	1.56	2.24	2.63	0.13
	Total	2.05	1.63	1.91	2.06	0.12
	SEM	0.20	0.07	0.40	0.21	
C 15:0 (Pentadecanoic)	0	0.35	0.20	0.22	0.39	0.03
	1 week	0.65	1.23	1.56	1.34	0.25
	2 weeks	0.98	0.80	0.75	0.63	0.18
	3 weeks	0.58	1.04	0.38	0.48	0.19
	Total	0.64	0.81	0.73	0.71	0.10
	SEM	0.12	0.23	0.25	0.19	
C 16:0 (Palmitic)	0	26.14	26.21	26.58	^y^ 26.08	0.28
	1 week	28.08 ^b^	27.83 ^b^	26.24 ^b^	^x^ 31.32 ^a^	0.58
	2 weeks	27.99 ^b^	27.19 ^b^	25.47 ^b^	^x^ 31.42 ^a^	0.68
	3 weeks	27.68	28.15	27.38	^y^ 26.55	0.34
	Total	27.47 ^b^	27.34 ^b^	26.42 ^b^	28.84 ^a^	0.27
	SEM	0.29	0.30	0.40	0.80	
C 17:0 (Heptadecanoic)	0	0.67	0.97	0.63	0.61	0.09
	1 week	0.45	0.56	0.49	0.51	0.02
	2 weeks	0.57	0.58	0.49	0.61	0.03
	3 weeks	0.54	0.61	0.72	0.67	0.04
	Total	0.56	0.68	0.58	0.60	0.03
	SEM	0.03	0.09	0.04	0.03	
C 18:0 (Stearic)	0	14.42 ^a^	^x^ 15.55 ^a^	11.40 ^b^	^x^ 13.04	0.52
	1 week	11.81 ^b^	^x^ 15.23 ^a^	14.82 ^a^	^y^ 11.52 ^b^	0.52
	2 weeks	11.92	^y^ 11.90	12.61	^y^ 11.74	0.31
	3 weeks	13.26	13.24	15.14	^x^ 14.72	0.33
	Total	12.85 ^b^	13.98 ^a^	13.49 ^ab^	12.75 ^b^	0.25
	SEM	0.32	0.50	0.56	0.55	
Total SFA	0	42.86	44.96	43.19	42.76	0.40
	1 week	43.64	46.94	46.02	47.95	0.55
	2 weeks	44.01	42.77 ^b^	41.48 ^b^	47.99 ^a^	0.88
	3 weeks	44.68	45.57	46.10	44.18	0.46
	Total	43.80 ^b^	45.06 ^ab^	44.19 ^b^	46.47 ^a^	0.32
	SEM	0.40	0.59	0.71	0.59	

n = 3 and for total value n = 12; *—beef marinated in 3% brine; ^a,b,c^—means with different letters in the same row differ at *p* ≤ 0,05; ^x,y^—means with different letters in the same column differ at *p* ≤ 0.05; SEM—standard error of the mean; SFA—saturated fatty acid.

**Table 7:** The share of MUFA in the lipids of marinated sous-vide beef.

Fatty Acids (% of the Sum of All FA)	Storage Time	Control *	Beef Marinated with the Kiwiberry Addition			SEM
			10%	20%	30%	
C14:1 (Myristoleic)	0	0.63	0.47	0.65	0.58	0.05
	1 week	0.62	0.76	0.25	1.24	0.21
	2 weeks	0.63	0.90	0.53	0.67	0.10
	3 weeks	0.95	0.51	0.35	1.49	0.28
	Total	0.71	0.66	0.44	0.99	0.09
	SEM	0.10	0.15	0.07	0.29	
C15:1cis (Pentadecenoic)	0	0.02	0.03	0.04	0.02	0.01
	1 week	0.10	0.05	0.00	0.01	0.04
	2 weeks	0.02	0.18	0.28	0.10	0.21
	3 weeks	0.37	0.65	0.08	0.21	0.13
	Total	0.17	0.37	0.30	0.13	0.07
	SEM	0.07	0.18	0.18	0.06	
C16:1 (Palmitoleic)	0	4.22	3.72	4.41	4.26	0.19
	1 week	4.53	3.93	3.83	4.63	0.20
	2 weeks	4.63	5.12	5.02	4.89	0.17
	3 weeks	4.24	3.74	3.85	3.61	0.18
	Total	4.40	3.99	4.28	4.35	0.11
	SEM	0.13	0.26	0.27	0.17	
C17:1cis (Heptadecenoic)	0	0.72	0.64	0.74	0.75	0.07
	1 week	1.02	0.77	0.83	1.03	0.04
	2 weeks	0.97	1.04	1.09	1.14	0.03
	3 weeks	0.91	0.92	0.75	0.84	0.03
	Total	0.91	0.84	0.85	0.94	0.03
	SEM	0.04	0.05	0.08	0.05	
C 18:1n-9cis (Oleic)	0	41.79 ^a^	39.76	39.68	37.24 ^b^	0.60
	1 week	42.48 ^a^	37.62 ^b^	37.99 ^b^	34.45 ^b^	0.89
	2 weeks	42.26 ^a^	41.42 ^a^	41.32 ^a^	35.57 ^b^	0.86
	3 weeks	41.63	40.04	40.0	39.84	0.35
	Total	42.04 ^a^	39.71 ^b^	39.74 ^b^	36.78 ^c^	0.37
	SEM	0.30	0.47	0.50	0.68	
C22:1n-9 (Erucic)	0	0.13	0.17	0.18	^x^ 0.77 ^a^	0.03
	1 week	0.00 ^b^	0.21 ^b^	0.22 ^b^	^y^ 0.20	0.11
	2 weeks	0.14	0.20	0.40	^x^ 0.55	0.06
	3 weeks	0.00	0.12	0.00	^y^ 0.00	0.02
	Total	0.06 ^b^	0.18 ^b^	0.20 ^ab^	0.38 ^a^	0.04
	SEM	0.04	0.03	0.05	0.11	
Total MUFA	0	47.50	44.82	45.71	43.06	0.62
	1 week	48.93 ^a^	^y^ 42.88	^y^ 43.13	42.14 ^b^	0.84
	2 weeks	48.67 ^a^	^x^ 49.35 ^a^	^x^ 49.44 ^a^	43.11 ^b^	0.91
	3 weeks	48.10 ^a^	45.98 ^b^	45.04 ^b^	46.00 ^b^	0.56
	Total	48.30 ^a^	45.76 ^b^	45.83 ^b^	43.58 ^c^	0.40
	SEM	0.25	0.80	0.85	0.59	

n = 3 and for total value n = 12; *—beef marinated in 3% brine; ^a,b,c^—means with different letters in the same row differ at *p* ≤ 0.05; ^x,y^—means with different letters in the same column differ at *p* ≤ 0.05; SEM—standard error of the mean; MUFA—monounsaturated fatty acids.

**Table 8:** The share of PUFA in the lipids of marinated sous-vide beef.

Fatty Acids (% of the Sum of All FA)	Storage Time	Control *	Beef Marinated with the Kiwiberry Addition			SEM
			10%	20%	30%	
C 18:2 n-6 (Linoleic, LA)	0	4.63 ^b^	4.82 ^ab^	4.75 ^b^	^x^ 6.64 ^a^	0.36
	1 week	3.25	4.26	4.35	^y^ 4.63	0.18
	2 weeks	3.15	3.74	4.07	^y^ 4.04	0.15
	3 weeks	2.96	4.35	3.40	^y^ 4.52	0.24
	Total	3.50 ^b^	4.30 ^ab^	4.22 ^b^	4.95 ^a^	0.15
	SEM	0.22	0.23	0.14	0.41	
C 18:3 n-3 (α-Linolenic, ALA)	0	0.51	0.61	0.67	0.88	0.04
	1 week	0.34	0.50	0.55	0.58	0.03
	2 weeks	0.37 ^b^	0.56	0.56	0.74 ^a^	0.06
	3 weeks	0.40 ^b^	0.71	0.56	0.86 ^a^	0.11
	Total	0.40 ^c^	0.60 ^b^	0.59 ^b^	0.76 ^a^	0.04
	SEM	0.03	0.03	0.04	0.10	
C 20:3n-6 (Eicosatrienoic)	0	0.81	0.8	0.80	0.86	0.04
	1 week	0.46	0.73	0.77	0.52	0.05
	2 weeks	0.53	0.57	^y^ 0.60	0.56	0.03
	3 weeks	0.49 ^b^	0.57 ^b^	^x^ 1.22 ^a^	0.47 ^b^	0.12
	Total	0.57 ^b^	0.68	0.85 ^a^	0.60 ^b^	0.04
	SEM	0.05	0.05	0.09	0.08	
C 20:4n-6 (Arachidonic)	0	2.55	2.82	3.24	2.78	0.15
	1 week	2.30	2.84	3.32	2.99	0.15
	2 weeks	2.41	1.87	2.50	2.44	0.13
	3 weeks	2.37	1.41	2.07	2.17	0.18
	Total	2.41	2.24	2.78	2.59	0.09
	SEM	0.10	0.21	0.21	0.17	
C 20:5 n-3 (Eicosapentaenoic—EPA)	0	0.48	0.35	0.54	0.31	0.04
	1 week	0.33	0.65	0.64	0.33	0.05
	2 weeks	0.27	0.41	0.31	0.31	0.05
	3 weeks	0.31	0.63 ^a^	0.40	0.20 ^b^	0.07
	Total	0.35 ^b^	0.51 ^a^	0.53 ^a^	0.29 ^b^	0.03
	SEM	0.05	0.06	0.03	0.03	
C 22:5n-3 (Docosapentaenoic)	0	0.58	0.65	0.88	0.62	0.06
	1 week	0.69	1.06	1.08	0.63	0.07
	2 weeks	0.50	0.62	0.73	0.71	0.04
	3 weeks	0.60	0.64	0.82	0.57	0.10
	Total	0.61	0.74	0.88	0.63	0.04
	SEM	0.05	0.08	0.07	0.08	
C 22:6n-3 (Docosahexaenoic—DHA)	0	0.08 ^b^	0.11	0.21 ^a^	0.09	0.02
	1 week	0.04 ^b^	0.12	0.14	^x^ 0.19 ^a^	0.02
	2 weeks	0.02	0.09	0.09	0.10	0.01
	3 weeks	0.08	0.13	0.09	^y^ 0.05	0.01
	Total	0.05 ^b^	0.11 ^a^	0.13 ^a^	0.11 ^a^	0.01
	SEM	0.01	0.01	0.02	0.02	
Total PUFA	0	9.64	10.21	11.10	12.18	0.45
	1 week	7.42	10.17	10.85	9.90	0.44
	2 weeks	7.32	7.87	9.08	8.89	0.37
	3 weeks	7.21	8.44	8.86	8.82	0.34
	Total	7.90 ^b^	9.17 ^a^	9.97 ^a^	9.95 ^a^	0.25
	SEM	0.41	0.38	0.44	0.51	

n = 3 and for total value n = 12; *—beef marinated in 3% brine; ^a,b^—means with different letters in the same row differ at *p* ≤ 0,05; ^x,y^—means with different letters in the same column differ at *p* ≤ 0.05; SEM—standard error of the mean; PUFA—polyunsaturated fatty acids.

## Data Availability

The original contributions presented in the study are included in the article, further inquiries can be directed to the corresponding author.

## Abbreviations

AA	Antioxidant activity
ABTS	2,2′-azinobis (3-ethylbenzthiazoline-6-acid)
AI	Atherogenicity index
ALA	α-Linolenic acid (C18:3n-3)
DM	Dry mass
DPPH	2,2-diphenyl-1-picryhydrazyl
FA	Fatty acid
FRAP	Ferric-reducing antioxidant power assay
FM	Fresh mass
GA	Gallic acid
h/H	hypocholesterolemic fatty acids/Hypercholesterolemic fatty acids ratio
LA	Linoleic acid (C18:2n-6)
LA/ALA	Linoleic acid/α-linolenic acid
MUFA	Monounsaturated fatty acids
PUFA	Polyunsaturated fatty acids
PUFA/SFA	Sum of all determined polyunsaturated fatty acids/sum of all determined saturated fatty acids
P/S	Selected polyunsaturated fatty acids (LA + ALA)/selected saturated fatty acids (lauric + myristic + palmitic)
SEM	Standard error of the mean
SFA	Saturated fatty acids
TC	Total carotenoids
TChl	Total chlorophylls (sum of chlorophylls a + b)
TE	Trolox Equivalent
TI	Thrombogenicity index
TP	Total polyphenols
